# DEFINITIONS MATTER WHEN DIAGNOSING MILD COGNITIVE IMPAIRMENT

**DOI:** 10.1093/geroni/igz038.424

**Published:** 2019-11-08

**Authors:** Carol Derby, Charles B Hall, Mindy Katz

**Affiliations:** Albert Einstein College of Medicine, Bronx, New York, United States

## Abstract

Prior studies have shown that when standard diagnostic criteria are applied, the majority of individuals diagnosed with aMCI do not progress to clinical dementia, with a much larger proportion reverting to normal cognition. This suggests that a prospective confirmation of aMCI diagnosis may improve the specificity of the classification. We examined the rates of aMCI reversion using two definitions: one based on a single annual assessment, and one requiring a diagnosis over two consecutive annual assessments within the population based Einstein Aging Study Cohort. Using the definition that used a single annual assessment resulted in 224 incident aMCI cases in 5,321 person years of follow-up, for an incidence rate of 4.21 cases per 100 person years. Requiring the confirmatory diagnosis resulted in only 94 incident aMCI cases in 5736 person years of follow-up, for an incidence rate of 1.64 cases per 100 person years. 41% of the persons diagnosed with aMCI using the single annual assessment were cognitively normal at the next follow-up. Only 14% of the persons diagnosed with incident aMCI using the definition requiring later confirmation ever returned to being cognitively normal. When the aMCI definition that required confirmation was used, a dramatic reduction in the incidence rate of aMCI was observed in persons born after 1930, similar to what has been reported in the same cohort for dementia, but there was no such difference for the definition based on a single annual assessment.

